# *H. pylori* infection confers resistance to apoptosis via Brd4-dependent *BIRC3* eRNA synthesis

**DOI:** 10.1038/s41419-020-02894-z

**Published:** 2020-08-21

**Authors:** Yanheng Chen, Donald Sheppard, Xingchen Dong, Xiangming Hu, Meihua Chen, Ruichuan Chen, Jayati Chakrabarti, Yana Zavros, Richard M. Peek, Lin-Feng Chen

**Affiliations:** 1 Department of Biochemistry, University of Illinois at Urbana-Champaign, Urbana, 61801 IL USA; 2 Fujian Key Laboratory for Translational Research in Cancer and Neurodegenerative Diseases, Institute for Translational Medicine, School of Basic Medical Sciences, Fujian Medical University, Fuzhou, 350122 China; 3 The State Key Laboratory of Cellular Stress Biology, School of Life Sciences, Xiamen University, Xiamen, 361101 China; 4 Present Address: Department of Pharmacology and Systems Physiology, University of Cincinnati, Cincinnati, 45267 OH USA; 5 Division of Gastroenterology, Department of Medicine and Cancer Biology, Vanderbilt University School of Medicine, Nashville, 37232 TN USA; 6 Present Address: Department of Cellular and Molevular Medicine, College of Medicine, University of Arizona-Tucson, Tucson, 85724 AZ USA; 7 Carle R. Woese Institute for Genomic Biology, University of Illinois at Urbana-Champaign, Urbana, 61801 IL USA

**Keywords:** Cell biology, Cell biology, Cell death, Cell death

## Abstract

*H. pylori* infection is one of the leading causes of gastric cancer and the pathogenicity of *H. pylori* infection is associated with its ability to induce chronic inflammation and apoptosis resistance. While *H. pylori* infection-induced expression of pro-inflammatory cytokines for chronic inflammation is well studied, the molecular mechanism underlying the apoptosis resistance in infected cells is not well understood. In this study, we demonstrated that *H. pylori* infection-induced apoptosis resistance in gastric epithelial cells triggered by Raptinal, a drug that directly activates caspase-3. This resistance resulted from the induction of cIAP2 (encoded by *BIRC3*) since depletion of *BIRC3* by siRNA or inhibition of cIAP2 via BV6 reversed *H. pylori*-suppressed caspase-3 activation. The induction of cIAP2 was regulated by *H. pylori*-induced *BIRC3* eRNA synthesis. Depletion of *BIRC3* eRNA decreased *H. pylori*-induced cIAP2 and reversed *H. pylori*-suppressed caspase-3 activation. Mechanistically, *H. pylori* stimulated the recruitment of bromodomain-containing factor Brd4 to the enhancer of *BIRC3* and promoted *BIRC3* eRNA and mRNA synthesis. Inhibition of Brd4 diminished the expression of *BIRC3* eRNA and the anti-apoptotic response to *H. pylori* infection. Importantly, *H. pylori* isogenic *cagA*-deficient mutant failed to activate the synthesis of *BIRC3* eRNA and the associated apoptosis resistance. Finally, in primary human gastric epithelial cells, *H. pylori* also induced resistance to Raptinal-triggered caspase-3 activation by activating the Brd4-dependent *BIRC3* eRNA synthesis in a CagA-dependent manner. These results identify a novel function of Brd4 in *H. pylori*-mediated apoptosis resistance via activating *BIRC3* eRNA synthesis, suggesting that Brd4 could be a potential therapeutic target for *H. pylori*-induced gastric cancer.

## Introduction

Prevention of apoptosis by bacterial pathogens has emerged as a new trait of bacterial pathogenesis^1^. Accumulating evidence indicates that the ability to inhibit apoptosis during infection provides the bacterial pathogens with a survival advantage for the establishment of infection^1^. *H. pylori* is a Gram-negative bacterium that colonizes the gastric epithelium, causing chronic gastric inflammations and other gastric diseases, including gastric cancer^2^. In addition to the induction of pro-inflammatory cytokines, infection-associated inhibition of apoptosis is one of the mechanisms by which *H. pylori* infection stimulates cell proliferation and induces gastric cancer^2^. The *H. pylori cag* pathogenicity island (cag PAI) encodes a type IV secretion system that injects the bacterial virulence factor CagA into host epithelial cells^3^. CagA has been demonstrated to be critical for *H. pylori*-associated gastric cancer since infection with CagA-positive *H. pylori* strains is associated with an increased risk for gastric cancer compared to infection with CagA-negative strains^4,5^. The role of CagA in the pro-inflammatory cytokine production has been heavily studied, its contribution to cell survival is much less clear. CagA has been shown to affect cell proliferation and survival independent of inflammation since over-expression of CagA in mice induces hyperplasia in the gastric mucosa and polyps in the glandular stomach without inducing inflammation^6^.

Cellular apoptosis is mediated by the extrinsic or the intrinsic apoptosis pathway. The extrinsic pathway is initiated by the ligation of death receptors with FasL (Fas ligand) or TRAIL (TNF-related apoptosis-inducing ligand) at the plasma membrane, leading to the activation of initiator caspase-8^7,8^. On the other hand, the intrinsic pathway is initiated with the release of cytochrome c from the mitochondria to form apoptosomes for the activation of initiator caspase-9^7,8^. The activated initiator caspase-8 or -9 in turn activates the downstream effector caspase-3 or -7, which uses the protease activity to degrade a wide array of cellular proteins or DNA and induces cellular apoptosis^7,8^. Many small molecules have been developed as therapeutics in cancer treatment to trigger extrinsic or intrinsic apoptosis pathways in cancer cells^9^. Among these apoptosis-inducing small molecules, Raptinal is able to rapidly induce cancer cell death by directly activating the effector caspase-3, bypassing the activation of initiator caspase-8 and -9^10^.

The inhibitor of apoptosis proteins (IAPs) are a family of structurally related proteins, including XIAP (X-linked IAP), cIAP1 (cellular inhibitor of apoptosis protein-1), cIAP2, and Survivin, that inhibit apoptosis by blocking the activation of effector caspases^11^. Modulating the expression and activity of IAPs contributes to the apoptosis resistance by certain bacterial pathogens^1^. For example, upregulation of cIAP1, cIAP2, and XIAP is required to maintain apoptosis resistance in *Chlamydia trachomatis*-infected epithelial cells^12^. Similarly, *N. gonorrhoeae* infection increases the NF-κB-dependent expression of cIAP1 and cIAP2 to inhibit apoptosis^13^. Interestingly, *H. pylori* has also been shown to up-regulate cIAP2 expression in gastric epithelial cells and the gastric mucosa of *H. pylori* infected mice^14,15^. More importantly, cIAP2 is overexpressed in more than 70% of human gastric cancer tissues, and the expression levels of cIAP2 are higher in *H. pylori*-positive patient samples than in *H. pylori*-negative samples^15,16^. These studies highlight an important function of cIAP2 upregulation in *H. pylori* infection-mediated gastric diseases. However, the detailed mechanism for the upregulation of cIAP2 by *H. pylori* infection remains obscure.

The expression of IAPs has been reported to be regulated by the transcription factor NF-κB, which is an important regulator of the genes involved in innate and adaptive immune responses and for the survival and proliferation of certain cell types^17^. NF-κB is critically involved in *H. pylori*-induced pro-inflammatory cytokine expression and upregulation of cIAP2 in response to *H. pylori* infection^14,18,19^. NF-κB-dependent inflammatory gene expression is facilitated by the binding of various transcription regulators to the enhancers or promoters of NF-κB target genes^20^. Bromodomain-containing factor Brd4 has emerged as a key NF-κB co-activator by activating the positive transcription elongation factor b (P-TEFb) complex, which phosphorylates serine 2 of RNA polymerase II (RNAPII) for the efficient transcription elongation of NF-κB-dependent inflammatory genes^21,22^. In addition to its critical role in the synthesis of mRNA of inflammatory genes, emerging evidence indicates that Brd4 also facilitates NF-κB to stimulate the synthesis of enhancer RNA (eRNA) of inflammatory genes^23,24^. eRNAs are non-coding RNAs that are directed by enhancers and participate in enhancer function, affecting chromatin accessibility, chromatin looping, and the recruitment of transcription co-factors to the promoters^25^. Importantly, eRNAs have been implicated in the regulation of inflammatory transcription networks^24,26^. We have recently shown that Brd4 was involved in both *H. pylori*-induced *IL1* mRNA transcription and *IL1* eRNA synthesis^18^. However, whether Brd4-regulated eRNA synthesis is involved in the expression of cIAP2 in *H. pylori*-infected gastric epithelial cells remains to be determined.

To explore the mechanism by which *H. pylori* induces apoptosis resistance, we demonstrated that Brd4-mediated *BIRC3* eRNA synthesis was essential for *H. pylori*-induced *BIRC3* mRNA and cIAP2 protein expression. Brd4 and NF-κB were recruited to the enhancer region of *BIRC3* to regulate *BIRC3* eRNA and mRNA synthesis in a CagA-dependent manner. In addition, *H. pylori* infection also induced apoptosis resistance by activating the expression of *BIRC3* eRNA in primary human gastric epithelial cells.

## Results

### *H. pylori* infection confers resistance to Raptinal-induced apoptosis

**Fig. 1:**
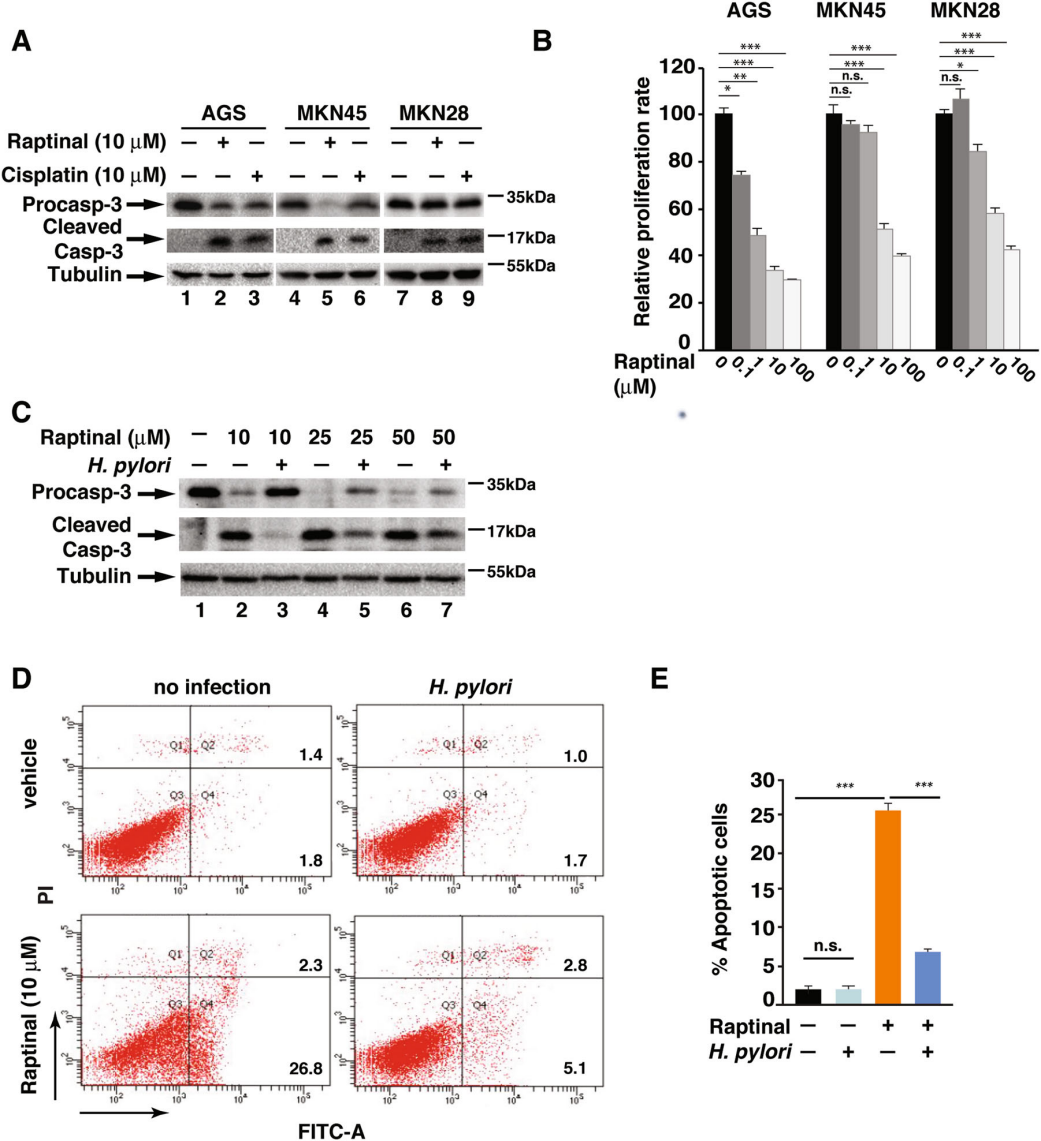
*H. pylori*-infected gastric epithelial cells are resistant to Raptinal-induced apoptosis. **a** Indicated cell lines were treated with 10 μM of Raptinal or Cisplatin for 2 or 24 h, respectively. Cell lysates were analyzed by immunoblotting for indicated proteins. **b** Indicated gastric cancer cell lines were treated with 10 µM of Raptinal for 72 h, and cell proliferation was measured by A490 nm using the CellTiter 96^R^AQueous One Solution cell proliferation assay (MTS) (Promega). **c** AGS cells infected with *H. pylori* G27 for 1 h followed by treatment with indicated concentration of Raptinal for 2 h. Cell lysates were analyzed by immunoblotting for indicated proteins. **d** AGS cells infected with *H. pylori* G27 for 1 h followed by treatment with Raptinal (10 µM) for 2 h. Apoptosis was assessed by flow cytometry using Annexin V-FITC/PI double staining assay. **e** The percentage of apoptotic cells from (**d**). All data are shown as the mean ± SD from three independent experiments. *p < 0.05, ***p < 0.001, n.s.: not significant.

To investigate the effect of *H. pylori* infection on apoptosis resistance in gastric epithelial cells, we first tested the ability of Raptinal to induce apoptosis in three different gastric epithelial cells, including AGS, MKN45, and MKN28. Raptinal has been shown to be able to induce apoptosis by activating caspase-3 within 30 min at a concentration of 10 μM^10^. Treatment with 10 μM of Raptinal for 2 h induced the cleavage of pro-caspase-3 into it’s active form in all three cell lines (Fig. 1a). Compared to Raptinal, the chemotherapeutic agent Cisplatin acted much slower. The activated caspase-3 was observed 24 h after 10-μM Cisplatin treatment with no cleaved caspase-3 appeared in 2 h (Fig. 1a and data not shown). These results suggest that Raptinal is a more effective apoptosis inducer than Cisplatin in gastric epithelial cells. Consistent with its ability to induce caspase-3 activation, Raptinal suppressed the proliferation of these gastric cancer cells (Fig. 1b).

We next examined the effect of *H. pylori* on Raptinal-induced caspase-3 cleavage. *H. pylori* G27 infection inhibited the Raptinal-induced caspase-3 cleavage at 10 μM, and this ability of *H. pylori* was still partially exerted with increasing concentrations of Raptinal (Fig. 1c). Since activation of caspase-3 is associated with cell apoptosis^10^, we then assessed the effect of *H. pylori* infection on Raptinal-induced apoptosis of gastric epithelial cells. Approximately 25% of AGS cells underwent apoptosis 2 h after Raptinal treatment (Fig. 1d, e) and infection with *H. pylori* G27 efficiently reduced the percentage of Raptinal-induced apoptotic cells (Fig. 1d, e). Altogether, these data demonstrate that *H. pylori* suppressed Raptinal-induced apoptosis through inhibition of caspase-3 activation.

### cIAP2 expression is required for *H. pylori* infection-induced apoptosis resistance in gastric epithelial cells

**Fig. 2:**
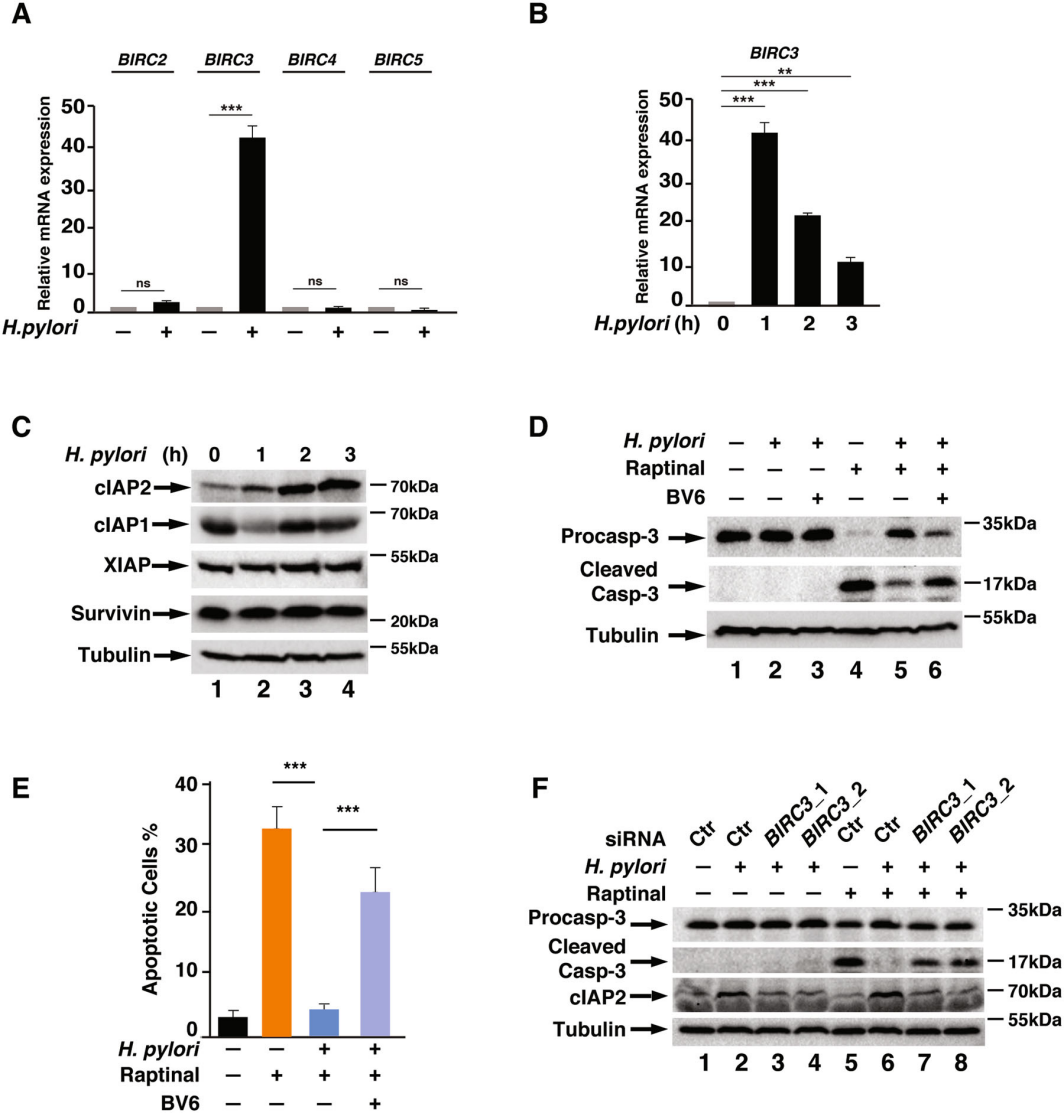
cIAP2 is responsible for *H. pylori* infection-mediated apoptosis resistance. **a** AGS cells were infected with *H. pylori* G27 for 1 h, and the expression of indicated genes was analyzed by qRT-PCR. **b**, **c** AGS cells were infected with *H. pylori* G27 for the indicated time points. mRNA levels of *BIRC3* were analyzed by qRT-PCR (**b**) and cell lysates were analyzed by immunoblotting for indicated proteins (**c**). **b** AGS cells pre-treated with BV6 (5 µM) for 1 h were infected with *H. pylori* G27 for another 1 h, followed by treatment with Raptinal (10 µM) for 2 h. Levels of indicated proteins were analyzed by immunoblotting, *n* = 3. **e** Percentage of apoptotic cells from (**c**) was analyzed by flow cytometry with Annexin V-FITC/PI double staining. **f** AGS cells transfected with two different siRNAs against *BIRC3* or control siRNA were infected with *H. pylori* G27 for 1 h, followed by treatment with Raptinal (10 µM) for 2 h. Levels of indicated proteins were analyzed by immunoblotting. All data are shown as the mean ± SD from three independent experiments. **p < 0.01, ***p < 0.001, n.s.: not significant.

IAPs, including XIAP and cIAP1 and cIAP2, are the primary proteins that inhibits caspase activation in apoptosis^11^. To understand the mechanism by which *H. pylori* infection induces apoptosis resistance, we measured the expression levels of these inhibitors of apoptosis in *H. pylori* infected AGS cells. Infection of AGS cells with *H. pylori* for 1 h dramatically increased the expression of *BIRC3* (gene of cIAP2), but not the expression of *BIRC2* (gene of cIAP1)*, BIRC4* (gene of XIAP), *BIRC5* (gene of Survivin) and other apoptosis related genes (Figs. 2a, b and S1). The levels of cIAP2 also increased with *H. pylori* infection in a time-dependent manner (Fig. 2c). There was no significant change for the protein levels of cIAP1, XIAP, and Survivin during the course of infection (Fig. 2c).

To determine the role of cIAP2 in *H. pylori* infection-induced apoptosis resistance, we treated the AGS cells with BV6, a known inhibitor of IAPs^27^, and examined the caspase-3 activation induced by Raptinal. Similar to Fig. 1c, Raptinal-induced caspase-3 activation in AGS cells and the caspase-3 activation was inhibited by *H. pylori* infection (Fig. 2d). However, this effect was reduced upon the addition of BV6 (Fig. 2d), indicating that inhibition of IAPs blocked *H. pylori* infection-induced apoptosis resistance. Consistently, more cells underwent apoptosis in *H. pylori*-infected AGS cells after BV6 treatment (Fig. 2e). These data suggest that IAPs might play a pivotal role in apoptosis resistance in response to *H. pylori* infection. Since *H. pylori* infection only induced the expression of cIAP2 but not XIAP and cIAP1 (Fig. 2c), cIAP2 was most likely responsible for the *H. pylori*-mediated apoptosis resistance. We next knocked down the expression of cIAP2 using siRNA and observed increased caspase-3 cleavage after Raptinal treatment compared to the control cells upon *H. pylori* infection, similar to BV6-treated cells (Fig. 2f). Collectively, these data demonstrate that cIAP2 plays a critical role in *H. pylori* infection-induced resistance to apoptosis in gastric epithelial cells.

### *H. pylori* infection-induced resistance to apoptosis is mediated by BIRC3 eRNA

**Fig. 3:**
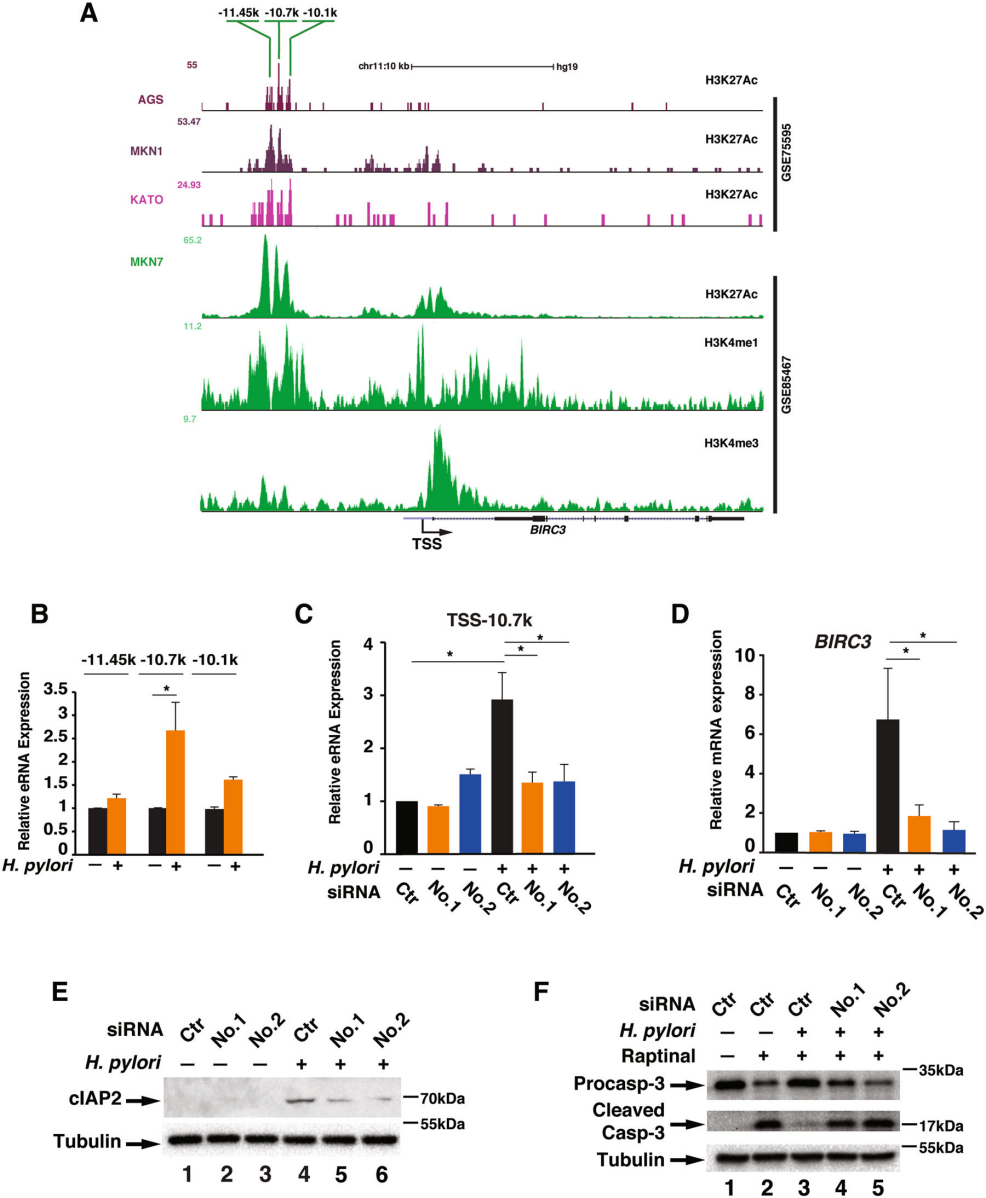
*H. pylori* stimulates the expression of *BIRC3* via eRNAs. **a** ChIP-seq profiles of two datasets (GSE75595 and GSE85467) for indicated histone marks of *BIRC3* locus in various gastric cancer cell lines (AGS, MKN1, KATO, and MKN7). H3K27Ac peaks are marked with their relative distance from the *BIRC3* TSS (Transcription Start Site). **b** AGS cells were infected with *H. pylori* for 1 h and the expression of eRNAs from each potential enhancer were analyzed by qRT-PCR. **c**, **d** AGS cells were transfected with two different siRNAs against eRNAs of *BIRC3* from −10.7 kb, then infected with *H. pylori* G27 for 1 h and the expression of indicated *BIRC3* eRNAs (**c**) and *BIRC3* mRNA (**d**) were analyzed by qRT-PCR. **e** AGS cells transfected with siRNAs against *BIRC3* eRNA as in (**d**) were infected with *H. pylori* G27 for 2 h and the cell lysates were immunoblotted for indicated proteins. **f** AGS cells transfected with two siRNAs against *BIRC3* eRNA as in (**d**) were infected with *H. pylori* for 1 h followed by treatment with Raptinal (10 µM) for 2 h. Cell lysates were immunoblotted for indicated proteins. All data are shown as the mean ± SD from three independent experiments. *p < 0.05.

Since *H. pylori*-induced cIAP2 expression is critical for apoptosis resistance, we next investigated how *H. pylori* stimulated the expression of *BIRC3*. We first determined the promoter and enhancer elements of *BIRC3* located on Chromosome 11 in human cells. Promoters and enhancers are often defined by unique histone marks with H3K27Ac associated with the active transcription of enhancers and promoters, H3K4Me3 associated with the promoters and H3K4me1 associated with active enhancers^28^. We examined the available chromatin immunoprecipitation-sequencing (ChIP-seq) datasets for histone marks in various gastric cancer cell lines, and found that H3K27Ac was enriched around the −10 kb region, upstream of TSS and the promoter region of *BIRC3* (Fig. 3a). In AGS cells, H3K27Ac is largely enriched in the enhancer region from −11.45 to −10.1 kb region but much less in the promoter region (Fig. 3a), suggesting that enhancers could be the major regulatory *cis*-element in the expression of *BIRC3*. Recent studies suggest that enhancer regulates gene expression via enhancer-derived eRNA, and we have previously showed that *H. pylori* induces eRNA synthesis for the efficient expression of inflammatory cytokine mRNAs such as *IL1A*^18,29^. In order to determine whether H3K27Ac enriched enhancer regions regulates *BIRC3* expression via eRNA, we examined the levels of eRNAs from several peaks containing enriched H3K27Ac, including −11.45, −10.7 and −10.1 k, with or without *H. pylori* infection (Fig. 3a). Of the three tested regions, *H. pylori* infection stimulated the expression of eRNAs significantly from −10.7 k region in AGS cells (Fig. 3b).

Since the function of eRNAs has largely been implicated in the regulation of its downstream mRNA, we next determined whether eRNAs from −10.7 k region was involved in the synthesis of *BIRC3* mRNA in response to *H. pylori* infection. We designed two different siRNAs targeting the −10.7 k eRNAs to inhibit *H. pylori*-induced expression of eRNAs of *BIRC3*. These two siRNAs efficiently suppressed *H. pylori*-induced eRNA synthesis (Fig. 3c). Importantly, inhibition of −10.7 k eRNA not only suppressed the expression of *BIRC3* mRNA but also the production of cIAP2 protein in response to *H. pylori* infection (Fig. 3d, e). We also assessed the effect of *BIRC3* eRNA on the activation of caspase-3. Inhibition of *BIRC3* eRNA synthesis by siRNAs reversed *H. pylori*-suppressed caspase-3 activation (Fig. 3f). All together, these results indicate that *BIRC3* gene expression is tightly regulated by *BIRC3* eRNAs, and this eRNA synthesis is critical for the *H. pylori*-mediated cIAP2 induction and caspase-3 inactivation.

### Brd4 is essential for *BIRC3* eRNA synthesis and *H. pylori* infection-mediated apoptosis resistance

**Fig. 4:**
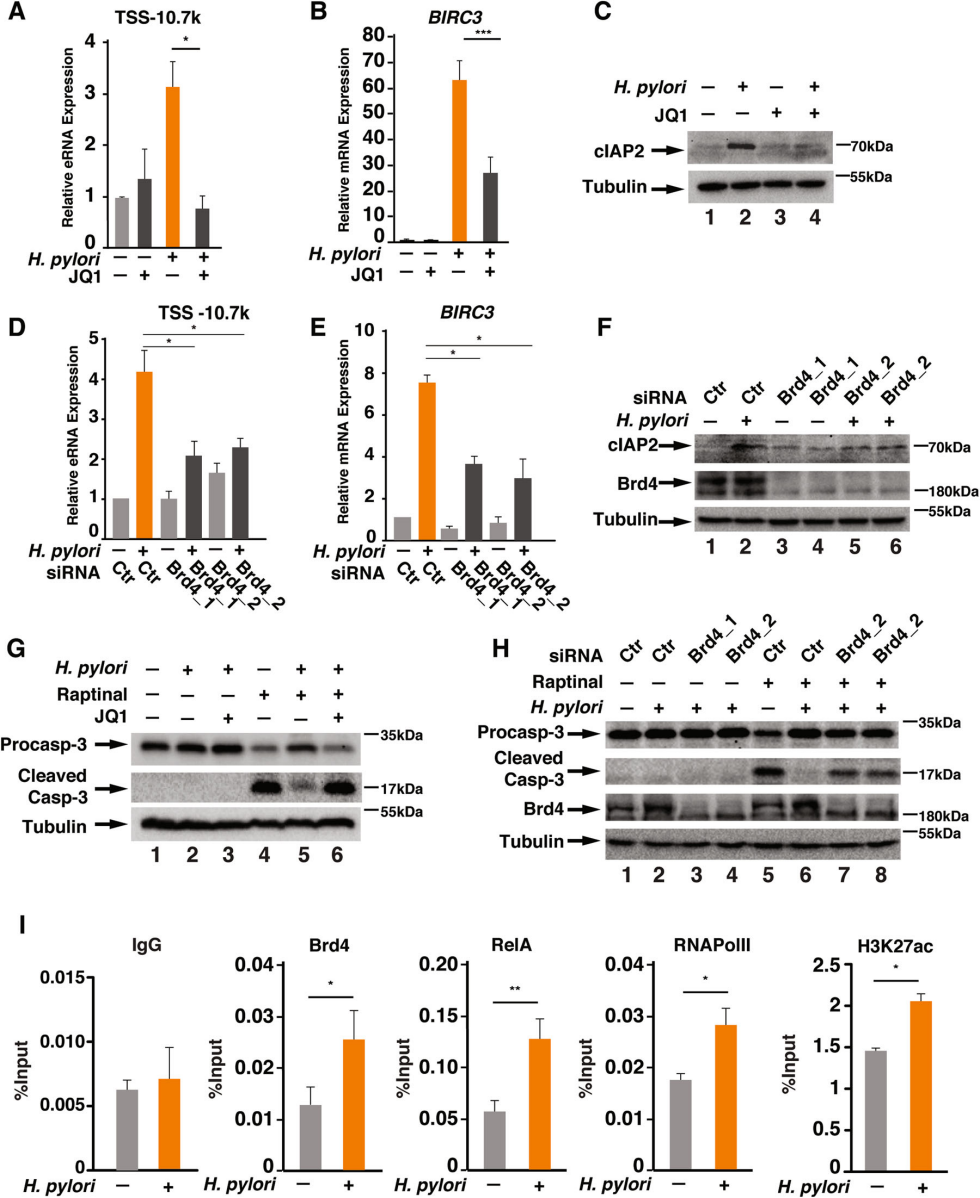
Brd4 facilitates the synthesis of *BIRC3* mRNA and eRNAs. **a**, **b** AGS cells were treated with or without 5 μM JQ1 as indicated for 1 h, followed by *H. pylori* infection for 1 h. The expression of *BIRC3* eRNAs (**a**) and mRNA (**b**) was analyzed by qRT-PCR. **c** AGS cells were treated with or without 5 μM JQ1 for 1 h, followed by *H. pylori* infection for 2 h. Cell lysates were immunoblotted for the indicated proteins. **d**, **e** AGS cells transfected with two different siRNAs against Brd4 were infected with *H. pylori* for 1 h. Expression of *BIRC3* eRNAs (**d**) and mRNA (**e**) was analyzed by qRT-PCR. (**f**) AGS cells transfected with siRNAs against Brd4 as in (**d**, **e**) were infected with *H. pylori* for 2 h. Cell lysates were immunoblotted for indicated proteins. **g** AGS cells were pre-treated with or without 5 μM JQ1 for 1 h as indicated. Cells were then infected with *H. pylori* for 1 h followed by treatment with Raptinal (10 µM) for 2 h. Cell lysates were immunoblotted for indicated proteins. **h** AGS cells transfected with siRNAs against Brd4 as in (**d**, **e**) were infected with or without *H. pylori* for 1 h followed by treatment with Raptinal (10 µM) for 2 h. Cell lysates were immunoblotted for indicated proteins. **i** AGS cells were infected with or without *H. pylori* G27 for 1 h. ChIP assays were performed using antibodies against Brd4, RelA, RNAPII and H3K27Ac and probed for the −10.7 k enhancer of *BIRC3*. All data are shown as the mean ± SD from three independent experiments. *p < 0.05, **p < 0.01, ***p < 0.001.

Recent studies reveal that Brd4 is a key mediator in the eRNA synthesis by binding to the enhancers^23^. We have also demonstrated that Brd4 is essential for the synthesis of *IL1* eRNA in response to *H. pylori* infection^18^. We next determined whether Brd4 was involved in the synthesis of *BIRC3* eRNA. We first treated AGS cells with JQ1, an inhibitor of Brd4 and other BET family proteins^30,31^ and found that JQ1 inhibited both *H. pylori*-induced *BIRC3* eRNA and mRNA synthesis (Fig. 4a, b). JQ1 also inhibited *H. pylori*-induced cIAP2 expression (Fig. 4c). To further confirm the involvement of Brd4 in the expression of *BIRC3* eRNA and mRNA, we employed siRNAs to deplete Brd4 and other two BET proteins, including Brd2 and Brd3, and measured *H. pylori*-mediated *BIRC3* expression. Depletion of Brd4 by siRNAs suppressed *H. pylori*-induced *BIRC3* eRNA and mRNA expression (Fig. 4d, e). More importantly, depletion of Brd4 also inhibited *H. pylori*-induced cIAP2 expression (Fig. 4f). Further supporting the role of Brd4 in regulating *H. pylori*-induced apoptosis resistance, *H. pylori* failed to suppress Raptinal-induced caspase-3 activation in JQ1 treated or Brd4 knockdown cells (Fig. 4g, h). However, depletion of Brd2 or Brd3 barely affected *H. pylori*-induced *BIRC3* mRNA and eRNA synthesis and cIAP2 expression (Figs. S2A–C) and did not affect *H. pylori*-induced apoptosis resistance (Fig. S2D). Collectively, these results demonstrate that Brd4 but not Brd2 or Brd3 is critically involved in *H. pylori-*induced cIAP2 induction and apoptosis resistance.

To further understand how Brd4 regulates *BIRC3* eRNA synthesis, we employed ChIPs to determine whether Brd4 is recruited to the enhancer region of *BIRC3*. Compared to uninfected cells, the binding of Brd4 to −10.7 enhancer region was significantly enhanced by *H. pylori* infection (Fig. 4i). NF-κB is known to assist the recruitment of Brd4 to the promoters or enhancers to activate CDK9 to facilitate RNAPII-dependent transcription elongation^21^. Consistently, the RelA subunit of NF-κB and RNAPII were also found to be enriched on the −10.7 k enhancer region after *H. pylori* infection (Fig. 4i). Similarly, H3K27Ac, the histone mark for active transcription, was also enriched in *H. pylori* infected cells (Fig. 4i). These ChIP results further demonstrate that *H. pylori* stimulates the recruitment of Brd4, likely through NF-κB, to the enhancer of *BIRC3* for the RNAPII-mediated *BIRC3* eRNA synthesis.

### *H. pylori* infection stimulates the expression of cIAP2 and apoptosis resistance in a CagA-dependent manner

**Fig. 5:**
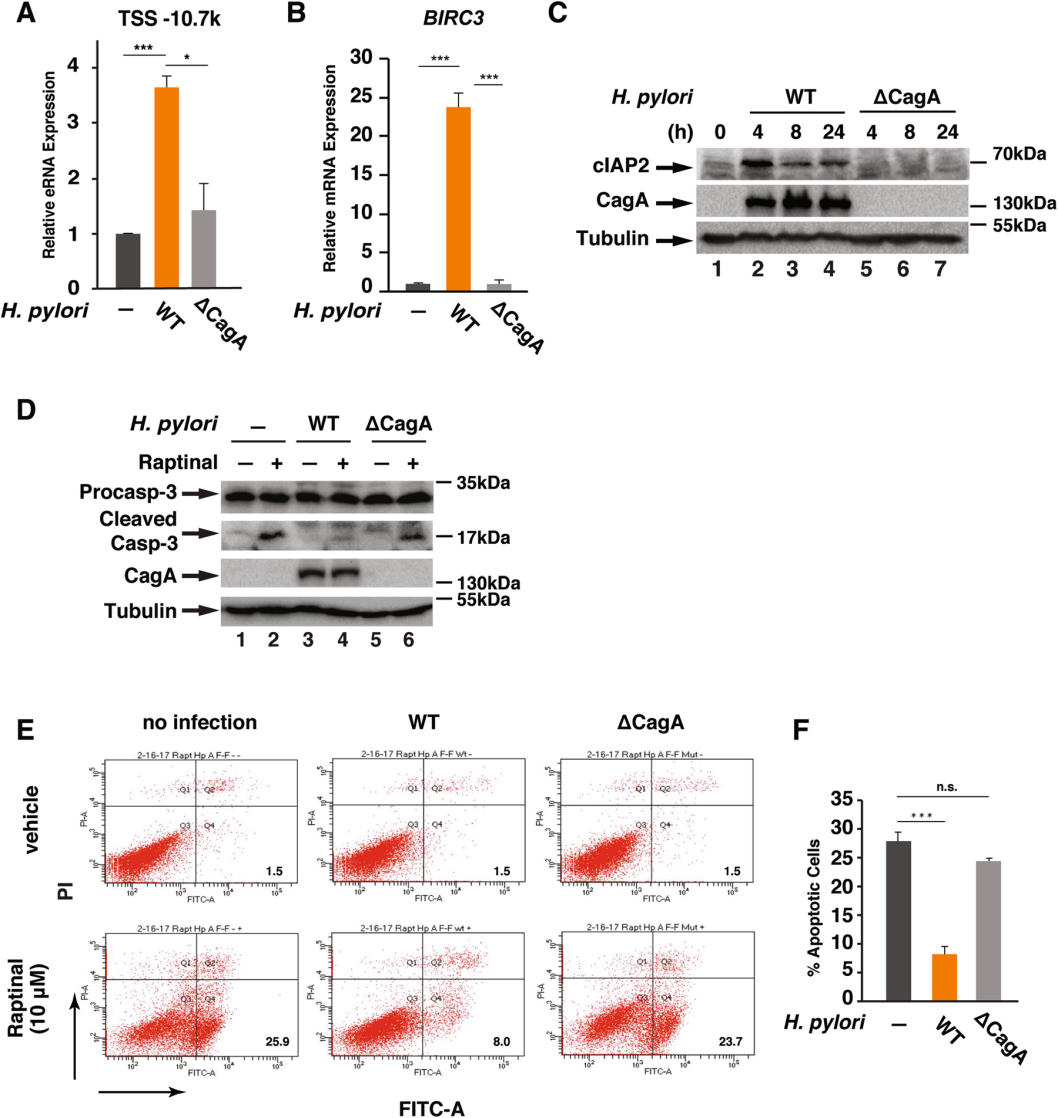
CagA is essential for *H. pylori*-induced *BIRC3* expression and apoptosis resistance. **a**, **b** AGS cells were infected with wild-type *H. pylori* G27 or the *cagA*-deficient isogenic mutant for 1 or 2 h for the expression of *BIRC3* eRNA (**a**) or mRNA (**b**), respectively. Levels of *BIRC3* eRNA and mRNA were analyzed by qRT-PCR. **c** AGS cells were infected with *H. pylori* G27 or *cagA*-deficient isogenic mutant for the indicated time points. Cell lysates were analyzed by immunoblotting for indicated proteins. **d** AGS cells were infected with WT *H. pylori* G27 or *cagA*-deficient isogenic mutant for 1 h, followed by the treatment with Raptinal (10 µM) for 2 h. Cell lysates were immunoblotted for the indicated proteins. **e** AGS cells infected with WT *H. pylori* G27 or *cagA*-deficient isogenic mutant for 1 h followed by the treatment with Raptinal (10 µM) for 2 h. Apoptotic cells was analyzed by FACS with Annexin V-FITC/PI staining. **f** The percentage of apoptotic cells from (**e**) for Raptinal-treated samples. All data are shown as the mean ± SD from three independent experiments. *p < 0.05, ***p < 0.001, n.s.: not significant.

Translocation of *H. pylori* virulent factor CagA into host cells induces higher levels of cytokine production by activating NF-κB and AP-1^32^. Due to the essential role of NF-κB in the expression of cIAP2^19,33^, we next investigated whether CagA was involved in the *BIRC3* eRNA and mRNA synthesis. Unlike WT *H. pylori, cagA*-deficient isogenic mutant failed to activate the expression of *BIRC3* eRNA and mRNA (Fig. 5a, b). Examining the cellular levels of cIAP2 in *H. pylori* infected AGS cells revealed that the expression of cIAP2 was upregulated by the infection in a CagA-dependent manner since infection of AGS cells with WT but not the *cagA*-deficient *H. pylori* enhanced the expression levels of cIAP2 (Fig. 5c).

Since cIAP2 is critical for the *H. pylori*-mediated inhibition of caspase-3 activation (Fig. 2), we next examined the effect of CagA on Raptinal-induced caspase-3 activation. Infection of cells with WT but not the *cagA*-deficient mutant *H. pylori* suppressed Raptinal-induced caspase-3 activation (Fig. 5d). We also examined the effect of CagA on Raptinal-induced cell apoptosis. While WT *H. pylori* suppressed Raptinal-induced cell apoptosis, the *cagA*-deficient isogenic mutant barely affected Raptinal-induced cell apoptosis (Fig. 5e, f). Collectively, these findings demonstrate that *H. pylori* CagA is essential for the induction of cIAP2 expression and *H. pylori*-infection-mediated apoptosis resistance.

### *H. pylori* induces cIAP2 expression and inhibits apoptosis in primary human gastric epithelial cells

**Fig. 6:**
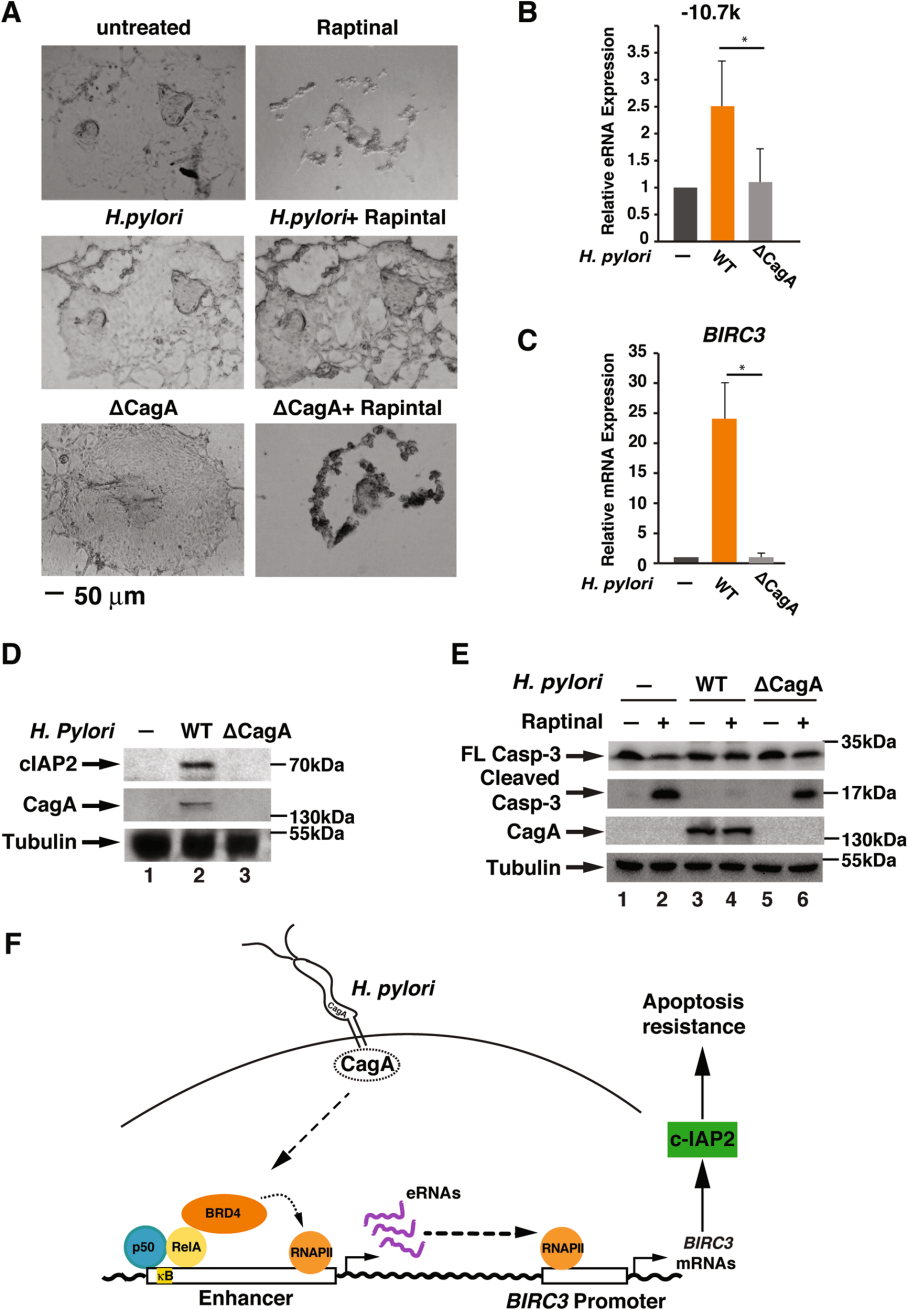
*H. pylori* induces cIAP2 expression and inhibits apoptosis in primary human gastric epithelial cells. **a** Representative images of primary human gastric epithelial cells with or without infection by wild-type *H. pylori* G27 or the *cagA*-deficient isogenic mutant for 1 h, followed by Raptinal treatment for 2 h. Scale bar = 50 μm. **b**, **c** Primary human gastric epithelial cells were infected with *H. pylori* G27 or *cagA*-deficient isogenic mutant for 1 h. Levels of *BIRC3* eRNA (**b**) or mRNA (**c**) were analyzed by qRT-PCR. **d** Primary human gastric epithelial cells were infected with *H. pylori* G27 or *cagA*-deficient isogenic mutant for 8 h. Cell lysates were immunoblotted for indicated proteins. (**e**). Primary human gastric epithelial cells were infected with *H. pylori* G27 or *cagA*-deficient isogenic mutant for 1 h followed by the treatment with Raptinal (10 µM) for 2 h. Cell lysates were immunoblotted for indicated proteins. **f** Schematic model for the *H. pylori* infection-mediated cIAP2 induction and apoptosis resistance. *H. pylori* stimulates the recruitment of Brd4 to the enhancer of *BIRC3* in a CagA-dependent manner. This recruitment facilitates RNAPII-mediated eRNA and mRNA synthesis and the induction of cIAP2, resulting in the inhibition of apoptosis. All data are shown as the mean ± SD from three independent experiments. *p < 0.05.

Gastrointestinal organoids closely recapitulate the cellular complexity of the organ tissue from which they are derived to mimic intact tissue in vivo^34,35^. Culture of primary human- and mouse-derived gastric organoids has emerged as a novel approach to study the pathogenesis of *H. pylori* infection in a more physiological setting^34,36–38^. We next assessed the effect of *H. pylori*-mediated apoptosis resistance in human gastric organoids. We acquired organoids derived from primary tissues and cultured them to form a 2D gastric epithelial cell monolayer (Fig. 6a). Treatment of the gastric epithelial cell cells with Raptinal dramatically changed the cell morphology, indicating Raptinal-induced cell death (Fig. 6a, upper panels). However, infection of cells with *H. pylori* prevented Raptinal-induced morphology changes (Fig. 6a, middle panels). Conversely, infection with the *cagA*-deficient mutant failed to prevent Raptinal-induced cell morphology changes (Fig. 6a, lower panels), indicating CagA-dependent apoptosis resistance by *H. pylori* infection. We next measured *H. pylori*-stimulated *BIRC3* eRNA and mRNA synthesis using these organoids. Similar to AGS cells, WT but not the *cagA*-deficient mutant *H. pylori* stimulated the expression of *BIRC3* −10.7 k eRNA and mRNA (Fig. 6b, c) and induced the cIAP2 expression (Fig. 6d). Furthermore, Raptinal also effectively induced the cleavage of caspase-3 in these organoids (Fig. 6e). Importantly, infection with WT but not the *cagA*-deficient mutant *H. pylori* suppressed Raptinal-induced caspase-3 activation in primary gastric epithelial cells (Fig. 6e). All together, these data demonstrate that *H. pylori* infection stimulates the expression cIAP2 in a CagA-dependent manner in human normal gastric epithelial cells, further supporting the notion that *H. pylori* infection confers apoptosis resistance in gastric epithelial cells.

## Discussion

*H. pylori* infection-associated chronic inflammation and apoptosis resistance contribute to the development of gastric diseases, including gastritis and gastric cancer^39^. *H. pylori*-mediated inflammation is associated with its ability to activate NF-κB and the subsequent recruitment of co-activator Brd4 to the promoters and enhancers to facilitate NF-κB-dependent inflammatory gene expression^18^. In this study, we found that Brd4 was also essential for *H. pylori*-induced NF-κB-dependent anti-apoptosis gene *BIRC3* expression (Fig. 6e). By regulating *BIRC3* eRNA and mRNA synthesis, Brd4 contributes to the development of apoptosis resistance in *H. pylori*-infected gastric epithelial cells (Fig. 6e).

We also showed that CagA was required for the *H. pylori*-induced cIAP2 expression and apoptosis resistance (Figs. 5 and 6). Since *H. pylori* CagA uses diverse pathways to activate NF-κB, which regulates the expression of cIAP2^14,19,32^, it is possible that CagA utilizes activation of NF-κB to regulate the expression of *BIRC3* eRNA and the production of cIAP2. In addition to its involvement in NF-κB-dependent expression of cIAP2 for apoptosis resistance, CagA appears to utilize other strategies to inhibit cell apoptosis in infected cells. Injection of CagA protein into gastric epithelial cells has been shown to counteract the apoptotic action of *H. pylori* VacA for the survival of *H. pylori* after colonization^40^. CagA-mediated pro-inflammatory cytokine expression and chronic inflammation could also provide a survival environment for infected gastric epithelial cells^41^. Furthermore, *H. pylori* CagA could activate ERK and anti-apoptotic protein MCL1 in the gastric pits to suppress apoptosis or subvert the apoptosis-stimulating protein p53^42,43^. How CagA coordinates these different cellular pathways to acquire the apoptosis resistance in *H. pylori*-infected cells remains to be further evaluated.

By activating the expression of cIAP2, *H. pylori* infection likely provides a survival advantage for the infected gastric epithelial cells, resulting in the enhanced colonization to establish long-term infection. Intriguingly, *H. pylori* can also induce epithelial cell apoptosis after prolonged infection. *H. pylori* activates the release of cytochrome c for the activation of caspase-8 and the apoptosis after 12 hr infection^44^. The induction of cIAP2 expression could be as early as 1 h after *H. pylori* infection (Fig. 2). The cell apoptosis after extended infection might result from reduced cIAP2 expression mediated by some negative feedback regulators of NF-κB (Fig. 5c). In this regard, *H. pylori* has been reported to activate A20, an inhibitor of NF-κB, to suppress NF-κB-mediated anti-apoptotic effect^45^. *H. pylori* infection could also induce cell death in macrophages by polyamine-dependent mechanisms and by signaling via ERK-MAPK pathway, enabling the *H. pylori* to escape immune cells for persistent infection^46–48^. It has to be noted that *H. pylori* has been shown to sensitize TRAIL- and FasL-mediated apoptosis in gastric epithelial cells^49,50^, although *H. pylori* infection conferred resistance to Raptinal-induced apoptosis (Fig. 1). It appears that *H. pylori* infection differentially affects the intrinsic and the extrinsic apoptosis pathways. Therefore, *H. pylori* infection-mediated cell death or cell survival depends on the cell types and the apoptosis pathways.

*H. pylori* has been shown to regulate the expression of cIAP2 by activating the promoter of *BIRC3* via NF-κB in a luciferase reporter assay^14^. In addition to the promoter-mediated transcription, our current study demonstrated that *BIRC3* enhancer was actively involved in the transcription of *BIRC3* mRNA. *H. pylori* stimulated the synthesis of enhancer-derived eRNAs, which were critical for the transcription of *BIRC3* mRNA and the production of cIAP2 (Fig. 3). eRNAs are regulatory non-coding RNAs that could regulate *cis* localized mRNA expression in various cell types^25,26^. eRNAs regulate gene transcription via enhancing the interaction between enhancers and promoters or facilitating the RNAPII recruitment and activation on the promoters of target genes^24,26^. Consistent with eRNA’s role in gene transcription, inhibition of the *BIRC3* eRNA synthesis abolished *BIRC3* mRNA synthesis and cIAP2 production (Fig. 3). Importantly, inhibition of eRNA synthesis also impaired *H. pylori*-mediated inhibition of caspase-3 activation in gastric epithelial cells (Fig. 3f), suggesting that modulation of *BIRC3* eRNA synthesis could dictate the survival and death outcome in *H. pylori* infected cells. It appears that cIAP2 is the major IAPs responsible for *H. pylori*-induced apoptosis resistance since the expression of *BIRC3* but not *BIRC2* and *BIRC4* was significantly upregulated by *H. pylori* infection (Fig. 2a, c) and inhibition of cIAP2 was sufficient to eliminate *H. pylori* infection-mediated apoptosis resistance (Fig. 2f). However, cIAP1 and XIAP might also contribute to *H. pylori* infection-associated apoptosis resistance since cIAP1, cIAP2, and XIAP tend to form stable IAP complexes to more efficiently block the activation of caspases^11^.

*H. pylori* infection stimulated the recruitment of Brd4 to the active *BIRC3* enhancer, which is associated with H3K27Ac marks (Fig. 4i). More importantly, Brd4 co-occupied with RelA and RNAPII on the eRNA-producing *BIRC3* enhancer and was responsible for *H. pylori*-induced *BIRC*3 eRNA synthesis (Fig. 4i). The recruitment of Brd4 and RNAPII to *BIRC3* enhancer likely relies on the binding of NF-κB to the enhancer since NF-κB is known to assist the recruitment of Brd4 and RNAPII to the enhancer regions of many genes^18^. Brd4 has emerged as a therapeutic target in cancer and inflammatory diseases, and there are many clinical trials with different BETi, including JQ1, for their efficacies against tumors and inflammation^51,52^. In this study, we demonstrated that JQ1 increased the sensitivity of gastric epithelial cells to Raptinal-induced apoptosis in *H. pylori*-infected cells (Fig. 4). In the presence of JQ1, *H. pylori* barely activated the expression of cIAP2 and failed to suppress Raptinal-induced caspase-3 activation (Fig. 4). Therefore, future studies might be needed to test whether JQ1-like small molecules could be potentially used in combinational therapy to increase the sensitivity of cancer cells to chemotherapy or reduce the chemoresistance by down-regulating the expression of anti-apoptosis genes.

Gastric organoids have emerged as a model system to study *H. pylori* pathogenesis^37^. In addition to the gastric cancer epithelial cells, we found that *H. pylori* also activated the expression of *BIRC3* eRNA and mRNA and suppressed Raptinal-induced caspase-3 activation in human gastric organoids (Fig. 6). Therefore, the mechanism we identified in this study for *H. pylori* infection-mediated apoptosis resistance might indeed represent the infected epithelial cells in vivo. Consistent with this, we found that the expression of *BIRC3* was enhanced in the stomach of *H. pylori*-infected mice, and the expression was inhibited by the treatment of JQ1 (data not shown).

In summary, we have identified a novel function of Brd4 in *H. pylori*-induced apoptosis resistance. Brd4 was critically involved in *H. pylori*-mediated apoptosis resistance by inducing the production of cIAP2 via *BIRC3* eRNA synthesis. Inhibition of Brd4 or elimination of Brd4-mediated *BIRC3* eRNA synthesis suppressed infection-associated apoptosis resistance. Many anti-cancer drugs induce cancer cell apoptosis during chemotherapy and chemoresistance often occurs due to the development of apoptosis resistance^53^. Identification of Brd4 as a novel regulator of *H. pylori*-induced apoptosis resistance provides not only new insights into the pathogenesis of *H. pylori* infection but also provides potential alternative approaches by targeting Brd4 for the prevention and treatment of *H. pylori*-associated gastric cancer and drug-resistance. In addition to *H. pylori*, various pathogens, including *Chlamydophila pneumonia, Neisseria gonorrhoeae* and influenza virus, induce cIAP2 expression for the apoptosis resistance and persistent infection^13,54,55^. It would be of great interest to determine whether inhibition of Brd4 could be a general approach to eliminate pathogen-infection-associated apoptosis resistance.

## Materials and methods

### Cell lines, reagents, and antibodies

Human gastric cancer cell lines AGS, MKN28, MKN45 were cultured in RPMI-1640 medium supplemented with 10% fetal bovine serum; primary human gastric organoids were prepared and cultured as previously described^56^. Raptinal was a gift from Dr. Hergenrother (UIUC). BV6 was kindly provided by Dr. Vucic (Genentech, Inc). JQ1 has been described previously^57^. Cisplatin was purchased from Sigma. Lipofectamine® RNAiMAX Transfection was purchased from Thermo Fisher. Antibodies against cIAP1 (sc-7943), RNAPII (sc-899), RelA (sc-372), CagA (sc-25766), Brd3 (sc-81202) were purchased from Santa Cruz Biotechnology; antibodies against caspase-3 (#9662), Brd2 (#5848), cIAP2 (#3130), XIAP (#14334), Survivin (#2808) were purchased from Cell Signaling Technology; antibodies against β-Tubulin (T5168) were purchased from Sigma; antibodies against H3K27ac (ab4729) were purchased from Abcam.

### *H. pylori* strains, *H. pylori* culture and infection

*H. pylori* G27, a *cag*PAI (cag pathogenicity island)-positive and virulence factor CagA-positive clinical isolate, and its isogeneic cagA-deficient strain have been previously described^58^. *H. pylori* was cultured in bisulphite-free Brucella broth supplemented with 10% fetal bovine serum and 5 μg/ml vancomycin at 37 °C in the presence of 10% CO_2_. *H. pylori* cultured overnight was added to AGS or primary human gastric epithelial cells for infection at a multiplicity of infection of 100.

### siRNA knockdown, immunoblotting, and quantitative real-time PCR analysis

siRNAs targeting *BIRC3* (hs.Ri.BIRC3.13.1, hs.Ri.BIRC3.13.2)*, Brd2* (hs.Ri.Brd2.13.2, hs.Ri.Brd2.13.3) *and Brd3* (hs.Ri.Brd3.13.1, hs.Ri.Brd3.13.2) and negative control siRNA (Cat#51-01-14-03) were purchased from IDT; siRNAs targeting *Brd4* (Cat#4427038, ID: S23901/S23902) were purchased from Ambion; siRNAs targeting *BIRC3* eRNA were designed and purchased from Dharmacon, sequences for the duplexes are No.1: GGAGAAAGAAUCUGUUUUAUU; UAAAACAGAUUCUUUCUCCUU, No.2: CAAGUUGGAUUGAACAUCAUU; UGAUGUUCAAUCCAACUUGUU. All siRNAs were transfected using Lipofectamine RNAiMAX Transfection Reagent (Invitrogen) according to the manufacturer’s protocol. Twenty-four hr post-transfection, cells were used for various experiments. Immunoblotting and quantitative real-time PCR analysis were performed as previously described^21^. All the western blots are representative images of at least three independent experiments. PCR primers for mRNA and eRNAs were purchased from Integrated DNA Technologies and the sequences of primers are provided in the Supplementary Information.

### ChIP-seq analysis

ChIP-seq datasets were obtained from GSE85467 and GSE75595. Reads were mapped to human genome (GRCh37/hg19) using bowtie2^59^. Peaks were identified using HOMER findPeaks program and annotated using anotatePeaks.pl^60^. Finally, the resulting data were uploaded to UCSC genome browser to obtain the visual mapping of each histone modification^61^.

### Chromatin immunoprecipitation assays (ChIPs)

ChIP assay was performed as previously described^57^. In brief, 10 to 20 million cells were fixed in 1% formaldehyde for 10 min and sonicated using a bioruptor (Cole-Parmer) to obtain DNA peak length at 500 bp, and lysates were immunoprecipitated overnight with various antibodies. Protein A agarose blocked with sheared salmon sperm DNA, was used to collect antibody-chromatin complexes. After reverse crosslink with proteinase K, DNA was extracted with DNA purification kit from Qiagen (Valencia, CA, USA). The sequence of ChIP primers are: −10.7 k forward: AAGCTACCTCTCAGCCTACTTT; −10.7 k reverse: CCACTGTTTTCTGTACCCGGA.

### Flow cytometry

Cell death was analyzed by fluorescence activated cell sorting with FITC-conjugated annexin V and propidium iodide (BD Bioscience) according to the manufacturer’s instructions. In brief, the cells were collected and washed followed by incubation with annexin V and PI for 15 min. Cells then were analyzed using BD LSR II flow cytometer. Flowjo software was used to analyze the data.

### Statistical analysis

Data analysis was carried out using GraphPad Prism version 5 (GraphPad Software, San Diego California USA). All data are presented as mean ± SD unless otherwise stated. Student unpaired *t*-test was used to analyze the data. For all data, *p* value ≤ 0.05 was considered statistically significant. *p < 0.05, **p < 0.01, and ***p < 0.005.

## Supplementary information

Supplementary Figure legends and methods

Supplementary Figure 1

Supplementary Figure 2
