# Supplementary material for: H. pylori infection confers resistance to apoptosis via Brd4-dependent BIRC3 eRNA synthesis
Source: Cell Death Dis. 2020 Aug 21;11(8):667. doi: 10.1038/s41419-020-02894-z (PMC7441315; doi:10.1038/s41419-020-02894-z)
Supplement: Supplementary file 1 — Supplementary Figure legends and methods [file 41419_2020_2894_MOESM1_ESM.docx]

**Supplementary Figure legends:**

**Fig. S1.** AGS cells were infected with *H. pylori* G27 for 1 hr and the mRNA levels of various indicated apoptosis-related genes were analyzed by qRT-PCR. All data are shown as the mean±SD from three independent experiments. *: *p*<0.05, n.s.: not significant.

**Fig. S2.** (A&B) AGS cells transfected with siRNAs against *Brd2* or *Brd3* were infected with *H. pylori* G27 for 1 hr for the expression of *BIRC3* eRNA (A) or mRNA (B), respectively. Levels of *BIRC3* eRNA and mRNA were analyzed by qRT-PCR. (C) AGS cells transfected with siRNAs against *Brd2* or *Brd3* were infected with *H. pylori* G27 for 2 hr, cell lysates were immunoblotted for indicated proteins. (D) AGS cells transfected with siRNAs against *Brd2* and *Brd3* were infected with *H. pylori* G27 for 1 hr, followed by the treatment with Raptinal (10 µM) for 2 hr. Cell lysates were immunoblotted for indicated proteins. All data are shown as the mean±SD from three independent experiments. *: *p*<0.05, , ***: *p*<0.001, n.s.: not significant.

List of primer sequences of qRT-PCR.

| *BIRC2 F* | GAATCTGGTTTCAGCTAGTCTGG |
| --- | --- |
| *BIRC2 R* | GGTGGGAGATAATGAATGTGCAA |
| *BIRC3 F* | AAGCTACCTCTCAGCCTACTTT |
| *BIRC3 R* | CCACTGTTTTCTGTACCCGGA |
| *BIRC4 F* | ACCGTGCGGTGCTTTAGTT |
| *BIRC4 R* | TGCGTGGCACTATTTTCAAGATA |
| *BIRC5 F* | AGGACCACCGCATCTCTACAT |
| *BIRC5 R* | AAGTCTGGCTCGTTCTCAGTG |
| *-10.1k F* | CTGCTGCTAGAGGAAGGAACC |
| *-10.1k R* | CCTTCTGGTGGACGTTAGCTC |
| *-10.7k F* | GGCATCACGTCTTCAGAGACA |
| *-10.7k R* | CACATTGCATCTGATGTTCAATCC |
| *-11.45k F* | TTTCCCAGGTAGAAGTGGCAG |
| *-11.45k R* | CACTGATCTGGCGTTGCTG |
| *BCL-2 F* | GTGTGTGGAGAGCGTCAAC |
| *BCL2-R* | CCCAGCCTCCGTTATCCT |
| *BCL-XL F* | GAATGAACTCTTCCGGGATGG |
| *BCL-XL R* | GACTCACCAATACCTGCATCT |
| *c-FLIP F* | AACCCTCACCTTGTTTCG |
| *c-FLIP R* | AACTCAACCACAAGGTCCA |
| *NOXA F* | GCAGAGCTGGAAGTCGAGTG |
| *NOXA R* | GAGCAGAAGAGTTTGGATATCAG |
| *BID F* | CTCCGTGATGTCTTTCACAC |
| *BID R* | ACATCGAGCTTTAGCCAGTC |
| *BAD F* | CATCTTGAATATGGGCGGAAGTA |
| *BAD R* | TTCACACGCACCGGAAG |
| *BIM F* | CTGGTCTGCAGTTTGTTGGA |
| *BIM R* | GGTGGCTGCAAGAATCAAGT |
| *BAK F* | CATCAACCGACGCTATGACTC |
| *BAK R* | GTCAGGCCATGCTGGTAGAC |
| *BMF F* | AGGCTGATGTGTCTGTGATG |
| *BMF R* | TGGGAGTCTAATCCACTCTC |
| *BAX F* | AGTAACATGGAGCTGCAGAG |
| *BAX R* | CAGTTTGCTGGCAAAGTAGAAA |
| *PUMA F* | TGGAGGGTCCTGTACAATCT |
| *PUMA R* | CACCTAATTGGGCTCCATCTC |
| *BNIP 3 F* | GCCCGGGATGCAGGAGGAGA |
| *BNIP 3 R* | GAGCAGCAGAGATGGAAGGAAAAC |
