# Supplementary figures and images for: H. pylori infection confers resistance to apoptosis via Brd4-dependent BIRC3 eRNA synthesis
Source: Cell Death Dis. 2020 Aug 21;11(8):667. doi: 10.1038/s41419-020-02894-z (PMC7441315; doi:10.1038/s41419-020-02894-z)

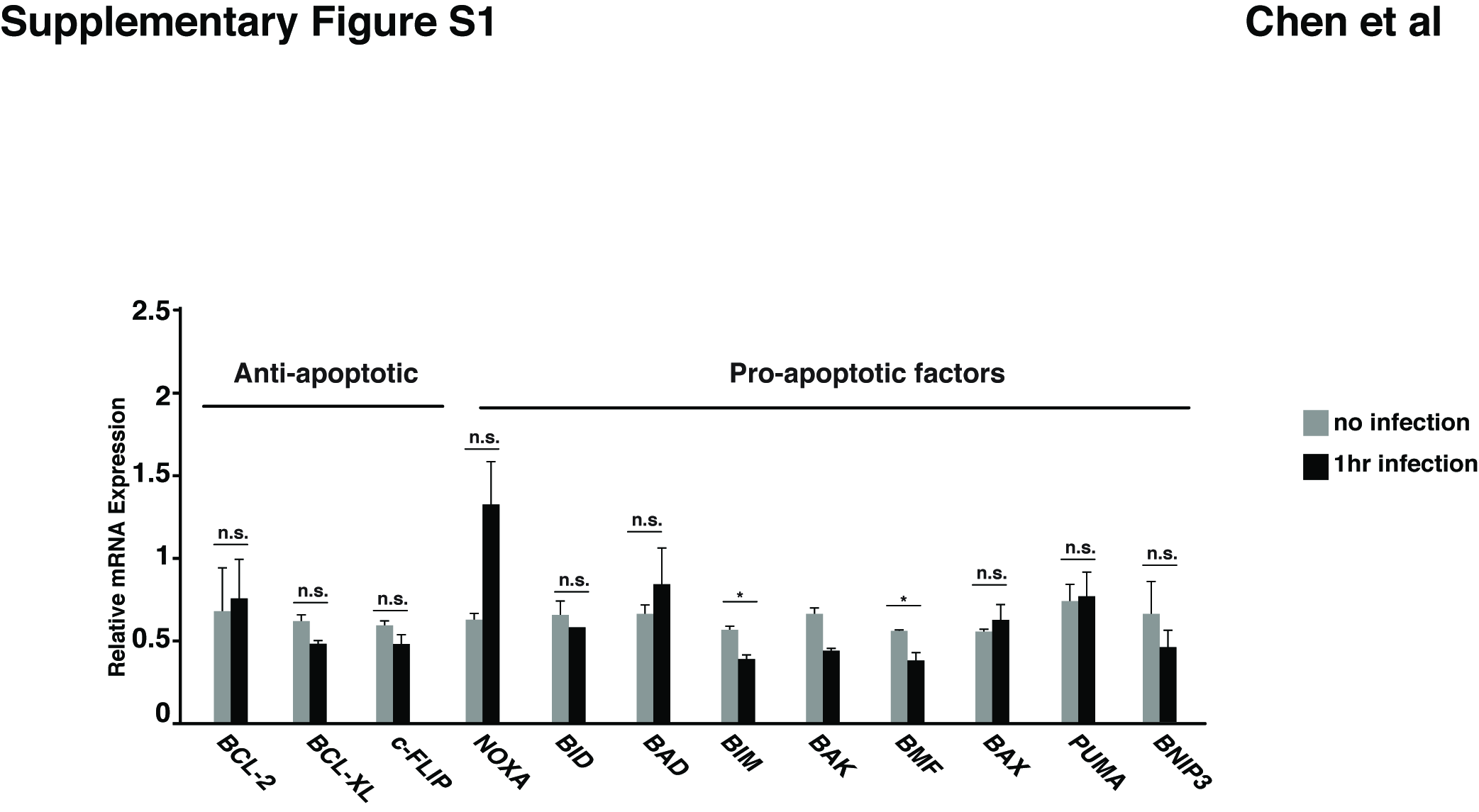

Supplement: Supplementary file 2 — Supplementary Figure 1 [file 41419_2020_2894_MOESM2_ESM.tif]

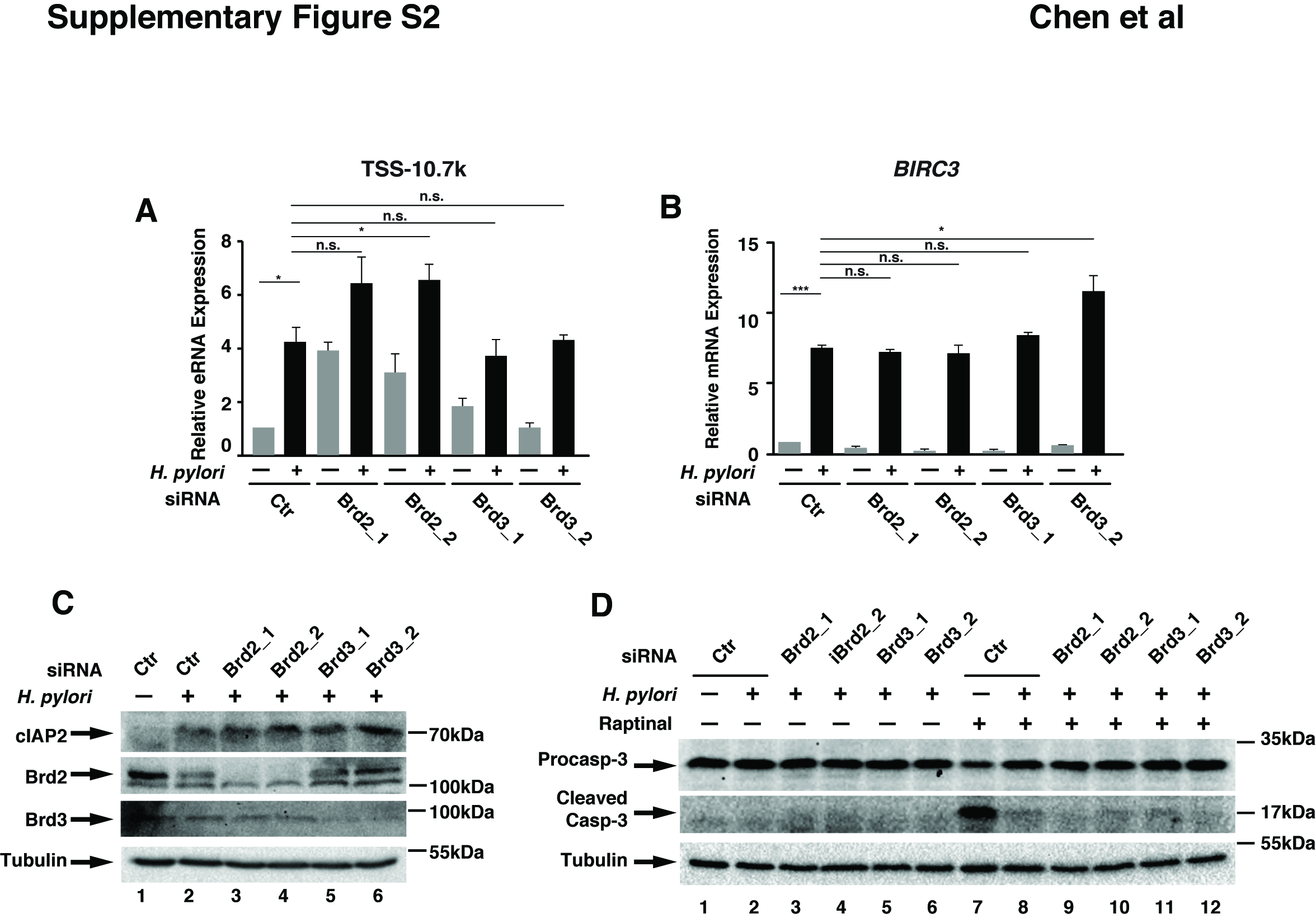

Supplement: Supplementary file 3 — Supplementary Figure 2 [file 41419_2020_2894_MOESM3_ESM.tif]
